# Accuracy of Wearable Transdermal Alcohol Sensors: Systematic Review

**DOI:** 10.2196/35178

**Published:** 2022-04-14

**Authors:** Eileen Brobbin, Paolo Deluca, Sofia Hemrage, Colin Drummond

**Affiliations:** 1 Department of Addictions King's College London London United Kingdom

**Keywords:** alcohol consumption, alcohol detection, alcohol monitoring, alcohol treatment, digital technology, ecologic momentary assessment, transdermal alcohol sensors, wearables, mobile phone

## Abstract

**Background:**

There are a range of wearable transdermal alcohol sensors that are available and are being developed. These devices have the potential to monitor alcohol consumption continuously over extended periods in an objective manner, overcoming some of the limitations of other alcohol measurement methods (blood, breath, and urine).

**Objective:**

The objective of our systematic review was to assess wearable transdermal alcohol sensor accuracy.

**Methods:**

A systematic search of the CINAHL, Embase, Google Scholar, MEDLINE, PsycINFO, PubMed, and Scopus bibliographic databases was conducted in February 2021. In total, 2 team members (EB and SH) independently screened studies for inclusion, extracted data, and assessed the risk of bias. The methodological quality of each study was appraised using the Mixed Methods Appraisal Tool. The primary outcome was transdermal alcohol sensor accuracy. The data were presented as a narrative synthesis.

**Results:**

We identified and analyzed 32 studies. Study designs included laboratory, ambulatory, and mixed designs, as well as randomized controlled trials; the length of time for which the device was worn ranged from days to weeks; and the analyzed sample sizes ranged from 1 to 250. The results for transdermal alcohol concentration data from various transdermal alcohol sensors were generally found to positively correlate with breath alcohol concentration, blood alcohol concentration, and self-report (moderate to large correlations). However, there were some discrepancies between study reports; for example, WrisTAS sensitivity ranged from 24% to 85.6%, and specificity ranged from 67.5% to 92.94%. Higher malfunctions were reported with the BACtrack prototype (16%-38%) and WrisTAS (8%) than with SCRAM (2%); however, the former devices also reported a reduced time lag for peak transdermal alcohol concentration values when compared with SCRAM. It was also found that many companies were developing new models of wearable transdermal alcohol sensors.

**Conclusions:**

As shown, there is a lack of consistency in the studies on wearable transdermal alcohol sensor accuracy regarding study procedures and analyses of findings, thus making it difficult to draw direct comparisons between them. This needs to be considered in future research, and there needs to be an increase in studies directly comparing different transdermal alcohol sensors. There is also a lack of research investigating the accuracy of transdermal alcohol sensors as a tool for monitoring alcohol consumption in clinical populations and use over extended periods. Although there is some preliminary evidence suggesting the accuracy of these devices, this needs to be further investigated in clinical populations.

**Trial Registration:**

PROSPERO CRD42021231027; https://www.crd.york.ac.uk/prospero/display_record.php?RecordID=231027

## Introduction

### Background

Current alcohol treatment largely relies on self-report data, which can be convenient and low-cost; however, there are limitations [1-3]. Reporting alcohol consumption over long periods may lead to recall bias [4]. Self-report can be made even more challenging when working with an alcohol-dependent population [5]. The current *gold standard* of alcohol assessments and alcohol research are self-report instruments, such as the Timeline Follow-Back [6] and the Alcohol Use Disorders Identification Test [7]. In recent years, various wearable transdermal alcohol sensor (TAS) devices have been developed. These devices measure alcohol consumption from alcohol vapors excreted through the skin via sweat, known as transdermal alcohol concentration (TAC), and can be worn on the wrist or ankle. Wearing a device all day for long periods allows for regular, repeated measurements and data capture in real time [8,9]. Thus, the TAS device is less likely to miss episodes of alcohol consumption than breath alcohol measurement. It is noninvasive, objective, and low-maintenance and allows behavior to be captured in real-world contexts [10]. These advantages address some of the limitations of other methods currently used, such as breathalyzers and blood and urine tests. Another advantage is that this technology has the potential to communicate with a smartphone [10-12]. Data captured can be uploaded over a mobile network and delivered to the patient, clinician, and researcher in near real time. This means reduced time and resources required by staff in addition to potentially more comprehensive and accurate data collected.

There is a range of TAS brands in various stages of development and validation, some available directly to the public. The 2 devices that have been most widely validated and reported on are SCRAM and WrisTAS [10,13-18]. More recently, Skyn from BACtrack, ION Wearable (rebranded as Proof) from Milo Sensors, and BARE and ORBIS from Smart Start have been developed. BACtrack uses a fuel cell alcohol sensor, whereas ION uses an enzymatic electrochemical biosensor cartridge [10,11]. BACtrack can be used as a stand-alone wearable or integrated into the band of a smartwatch [10,12] and, although it uses the same technology as WrisTAS, BACtrack uses a newer generation with improvements in performance and size [10]. At the beginning of 2021, Smart Start announced the development of ORBIS, a TAS with GPS monitoring technology designed to look like a smartwatch. Instead of fuel cell technology, this device uses a transdermal sensor that allows for continuous monitoring and has cellular communication embedded, removing the need to pair with a smartphone. This device is currently being tested through independent research and clinical and pilot trials [19]. These changes in TAS design and use—for example, linking to a smartphone app to display real-time data—address some of the limitations of the older devices. However, these new generations are in earlier stages of development, and researchers have only just begun exploring their validity, reliability, and usability [10-12].

The accuracy of these devices is evaluated by comparing TAC output with self-reported alcohol consumption via Timeline Follow-Back or drinking diaries, blood tests (blood alcohol concentration [BAC]), or breath alcohol concentration (BrAC). When comparing with self-report, the participant typically wears the device and notes how much alcohol they consume and when. This can allow for drinking in the participant’s own environment and for typically greater, self-dosing drinking to be recorded. However, there can be limits to self-report data, including accuracy of recall, adherence to recording, and social acceptability reporting bias, particularly in clinical populations [1-5]. Sensitivity, also known as true positive rate, measures the number of positive drinking events as indicated by TAC that are true positive drinking events when compared with self-report, BAC, or BrAC. Specificity, also known as true negative rate, measures the number of events where alcohol consumption was not detected and there was no alcohol consumed when compared with self-report, BAC, or BrAC [20,21]. Most studies report correlation or sensitivity and specificity data of the TAS device.

The data collected and calculated from TAC (peaks of use, time to peak, and area under the curve [AUC]) can be compared with the measurements collected via breathalyzer or blood tests (BrAC or BAC). Although TAS devices can automatically take readings at predefined time points, owing to the need for frequent administration of breathalyzer readings or the need for blood tests, studies using these comparisons typically require laboratory settings. This means that there are typically fixed-dose amounts of alcohol given by a research team, and the data are taken during a limited period (a few hours). TAC, BrAC, and BAC data are then statistically analyzed to determine the correlation. It would be optimal for TAS devices to perform with high accuracy in both laboratory and natural, real-world drinking situations.

van Egmond et al [15] conducted a systematic review in 2020 exploring the validity of wearable TAS devices. However, their review only included papers published since 2013 that used TAS devices and validation measures obtained from the devices and provided correlation or detection rate measures. This meant that papers identified by van Egmond that included Milo ION, MOX sensors, and a study on BACtrack were not included in their review [15] because of lack of validation measures. For our review, we decided to include all papers that met our inclusion criteria for investigating the use of wearable TAS devices, with no time constraints. We aimed to explore a broad overview of TAS technology within the field and demonstrate the growth in the range and development of devices.

### Objective

This systematic review aims to investigate the current knowledge by systematically identifying and evaluating the existing literature on the use of TAS devices in clinical and nonclinical populations, alone or in conjunction with a psychosocial intervention. The primary objective is to assess the level of accuracy of TAS devices. There is a linked review paper investigating the acceptability and feasibility of the devices (Brobbin et al, unpublished data, 2022).

## Methods

### Overview

This systematic review was conducted according to the PRISMA-P (Preferred Reporting Items for Systematic Reviews and Meta-Analyses Protocols) guidelines [22]. This protocol has been registered in PROSPERO (CRD42021231027). On review of the results, it was decided that the findings of the systematic review should be reported in 2 papers: one focusing on the accuracy outcomes and a second one focusing on the acceptability and feasibility outcomes.

### Inclusion Criteria

Studies meeting all criteria were included: full-text original studies published in peer-reviewed journals, written in English, and using a wearable transdermal sensor device reporting accuracy outcomes. For the purpose of this review, a wearable TAS is defined as a wearable device that can measure alcohol consumption from alcohol vapors excreted via the skin. There were no restrictions on publication year or participant clinical and demographic characteristics. Data based on conference abstracts, dissertations, and gray literature were not included.

### Information Sources

Bibliographic databases included CINAHL, Embase, Google Scholar, MEDLINE, PsycINFO, PubMed, and Scopus. Searches were carried out between February 1, 2021, and February 8, 2021 (Multimedia Appendix 1 [10,11,13,23-51] and Multimedia Appendix 2). The searches were supplemented by cross-checking the reference lists of key publications, related systematic reviews, and included papers.

All identified titles and abstracts were screened in Covidence (Veritas Innovation Ltd) to identify studies that potentially met the inclusion criteria. From this list, the full text was retrieved and assessed by a reviewer (EB); any doubts were discussed with a second reviewer (SH). Any disagreement was discussed with a third reviewer (PD). A data extraction form was created and pilot-tested with the first 5 included studies and refined as necessary (Multimedia Appendix 3). EB extracted the data independently, and the second reviewer (SH) completed the entry check for accuracy. Any discrepancies were resolved through discussion with the third reviewer (PD).

### Outcomes

All outcome measures reported, both objective and self-reported, were extracted. The primary outcome was the accuracy of wearable TAS devices for measuring alcohol consumption compared with other methods (self-report, BAC, and BrAC; definitions of these measures are reported in Multimedia Appendix 4 [14,23,25-28,32,37,52]). This included data on correlations, sensitivity and specificity, percentage or amount of unsuccessful data points collected, and any time lag differences for the sensor to reach peak TAC compared with peak BrAC and delay in peak time from drink consumption.

### Quality Assessment

We used the Mixed Methods Appraisal Tool (MMAT) as it is designed for appraisal in reviews that include a range of designs (qualitative, quantitative, and mixed methods) [53]. EB completed this independently, and any queries were discussed with a second reviewer (SH). Any disagreements were resolved through discussion (PD).

### Data Synthesis and Analyses

To draw conclusions about the included studies, we developed a synthesis of study characteristics. The data are summarized using a structured narrative description for accuracy measures, and we report the available data reported for correlations, sensitivity and specificity, failure rates, and time lag on SCRAM, WrisTAS, and BACtrack, with a separate section for other TAS model studies (with only 1 study on each of these). Not all studies reported accuracy data on all these measures. A meta-analysis was not possible because of the methodological heterogeneity.

## Results

### Overview

**Figure 1:**
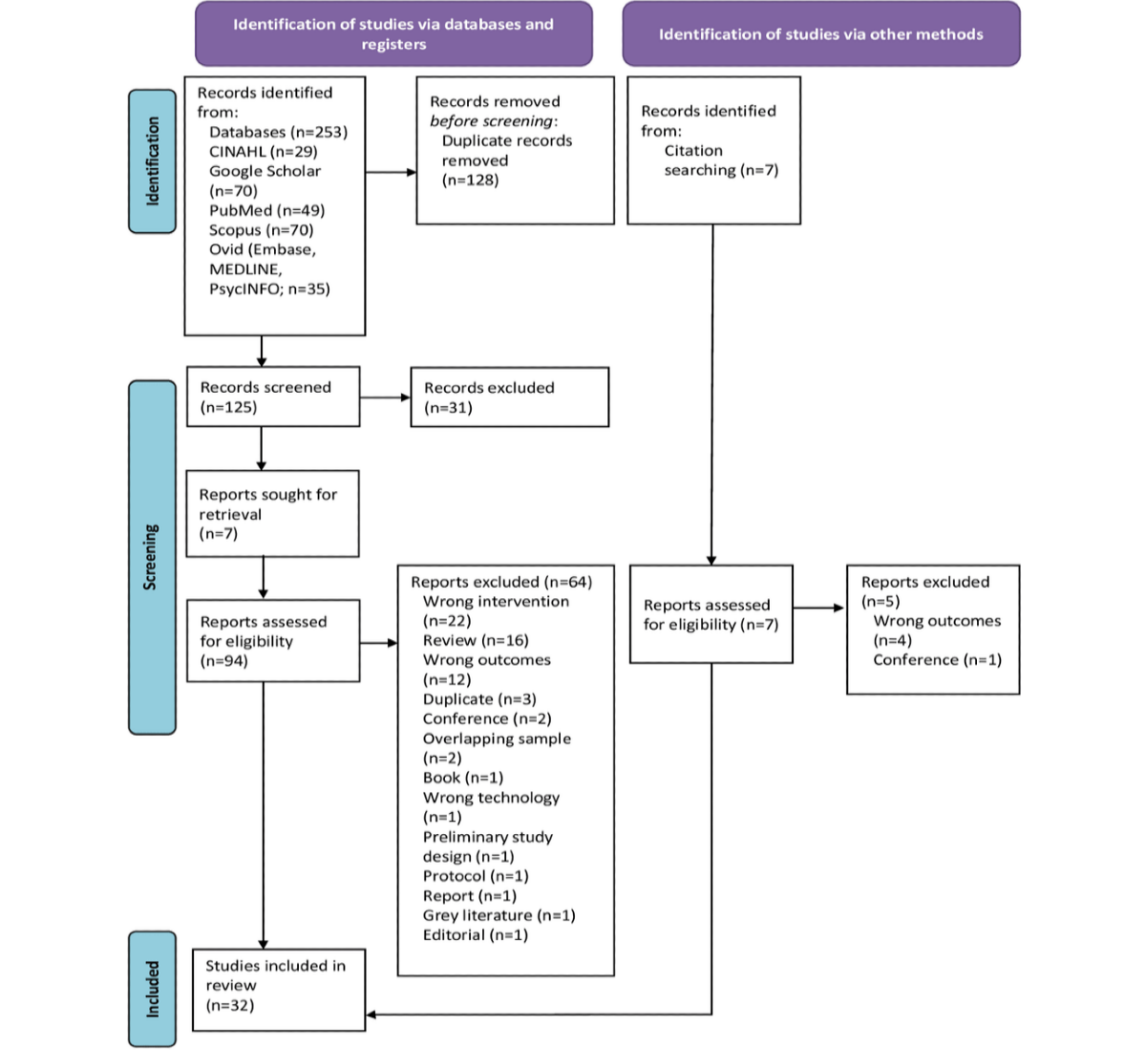
PRISMA (Preferred Reporting Items for Systematic Reviews and Meta-Analyses) flow diagram.

After removing duplicates, a total of 125 papers were screened; 31 (24.8%) were excluded at the title and abstract screening, and 94 (75.2%) full-text papers were assessed. Of those 94 papers, a total of 64 (68%) were then excluded (the reasons are provided in Multimedia Appendix 1). There were 7 additional papers identified through citation searching, of which 2 (29%) were included (Multimedia Appendix 1). The final sample included 32 publications (Figure 1).

### Study Characteristics

Of the 32 studies, 19 (59%) used a SCRAM device [13,23-40], 7 (22%) used a WrisTAS device [34,41-46], 5 (16%) used BACtrack Skyn [10,29,30,40,47], 1 (3%) used an ION Milo sensor [11], 1 (3%) used Quantac Tally [10], 1 (3%) used a wearable Internet of Things (IoT) sensor [48], 1 (3%) used an MOX sensor [49], 1 (3%) used a proton-exchange membrane (PEM) fuel cell sensor [50], and 1 (3%) did not name the TAS used [51]. Some studies (4/32, 13%) used more than one version of the device (eg, SCRAMx and SCRAM-II), and 16% (5/32) used more than one brand of device (Table 1).

A large proportion of studies (29/32, 91%) were conducted with adults in good health. Only 9% (3/32) of the studies included participants who were diagnosed alcohol-dependent clinical populations. Most of this research was conducted in the United States, with 94% (30/32) of the studies located there. The earliest paper included was from 1992, but most studies (21/32, 66%) were published as of 2015 (Table 1).

There were 1228 participants enrolled in total in the included studies, with 1147 included in the procedure or analysis. Therefore, 81 participants who were enrolled were not included in the results (for reasons such as withdrawing or missing data). In total, 1 paper (1/32, 3%) was still in the early stages of data collection for one of their studies and so these participant numbers were unknown [10]. Not all studies included detailed information on the participants’ age, sex, and ethnicity. From the information provided, it could be seen that the participants’ ages ranged from 18 to 58 years, most studies included women and men (27/32, 84%; 2/32, 6% included men only; 1/32, 3% included women only; and 2/32, 6% were unknown) and, for most studies (19/32, 59%), White participants represented a high proportion of the sample (11/32, 34% were unknown; Table 1).

**Table 1:** Characteristics of the included studies (N=32).

Study and year	Design	Aim	Participants enrolled (N=1228) vs participants included (n=1147), n^a^)	Population	Device	MMAT^b^ score
Alessi et al, 2019 [23]	Ambulatory	Assess how we can measure alcohol consumption with this technology	66 (63)	Clinical: alcohol outpatient	SCRAMx	40%
Ayala et al, 2009 [24]	Laboratory	Assessing nonalcoholic energy drinks with TAM^c^	15 (15)	Nonclinical: good health	SCRAM-II	80%
Barnett et al, 2017 [25]	RCT^d^, ambulatory	Effectiveness of TAM in implementing CM^e^ for alcohol reduction treatment in various population groups and evaluating the efficacy of CM reduction in alcohol use	30 (30)	Nonclinical: heavy drinkers	SCRAM-II and SCRAMx	80%
Barnett et al, 2014 [26]	Ambulatory	Assess how we can measure alcohol consumption with this technology	66 (66)	Nonclinical: heavy drinkers	SCRAM-II and SCRAMx	100%
Barnett et al, 2011 [27]	Ambulatory	Effectiveness of TAM in implementing CM for alcohol reduction treatment in various population groups and evaluating the efficacy of CM reduction in alcohol use	20 (13)	Nonclinical: heavy drinkers	SCRAM	80%
Bond et al, 2014 [41]	Ambulatory	Assess how we can measure alcohol consumption with this technology	250 (250)	Nonclinical: good health	WrisTAS (5, 6, and 7)	60%
Croff et al, 2020 [42]	Ambulatory	Assess acceptability, adherence, and feasibility with this technology	59 (57)	Nonclinical: good health	WrisTAS-7	80%
Davidson et al, 1997 [51]	Laboratory	Assess how we can measure alcohol consumption with this technology	15 (12)	Nonclinical: social drinkers	Not named	80%
Dougherty et al, 2012 [28]	Laboratory	Assess how we can measure alcohol consumption with this technology	22 (21)	Nonclinical: good health	SCRAM-II	100%
Fairbairn and Kang, 2019 [29]	Laboratory	Assess how we can measure alcohol consumption with this technology	50 (30)	Nonclinical: social drinkers	BACtrack prototype and SCRAM	60%
Fairbairn et al, 2020 [30]	Laboratory	Assess how we can measure alcohol consumption with this technology	110 (73)	Nonclinical: good health	BACtrack prototype and SCRAM	60%
Fairbairn et al, 2019 [13]	Mixed design	Estimating BrAC^f^ from TAC^g^	48 (48)	Nonclinical: social drinkers	SCRAM	100%
Hill-Kapturczak et al, 2014 [31]	Laboratory	Assess how we can measure alcohol consumption with this technology	22 (19)	Nonclinical: good health	SCRAM-II	80%
Jalal et al, 2020 [50]	Laboratory	Assess how we can measure alcohol consumption with this technology	8 (8)	Nonclinical: good health	PEM^h^ fuel cell sensor	60%
Karns-Wright et al, 2018 [32]	Ambulatory	Assess how we can measure alcohol consumption with this technology	32 (30)	Nonclinical: good health	SCRAM-CAM	80%
Karns-Wright et al, 2017 [33]	Laboratory	Estimating BrAC from TAC	61 (61)	Nonclinical: good health	SCRAM	80%
Lansdorp et al, 2019 [11]	Ambulatory	Assess how we can measure alcohol consumption with this technology	1 (1)	Nonclinical: good health	Milo sensor	60%
Lawson et al, 2019 [49]	Laboratory	Assess how we can measure alcohol consumption with this technology	6 (6)	Nonclinical: good health	MOX sensor	60%
Li et al, 2020 [48]	Laboratory	Assess how we can measure alcohol consumption with this technology	2 (2)	Nonclinical: good health	Wearable IoT^i^ alcohol sensor	80%
Luczak et al, 2015 [43]	Mixed design	Assess how we can measure alcohol consumption with this technology	32 (32)	Nonclinical: good health	WrisTAS-7	80%
Marques and McKnight, 2009 [34]	Ambulatory and laboratory	Assess how we can measure alcohol consumption with this technology	22 (22)	Nonclinical: good health	SCRAM and WrisTAS-5	60%
Norman et al, 2020 [35]	Ambulatory	Assess acceptability, adherence, and feasibility with this technology and how we can measure alcohol consumption with this technology	14 (14)	Nonclinical: good health	SCRAM	60%
Rash et al, 2019 [36]	Ambulatory	Assess how we can measure alcohol consumption with this technology	22 (19)	Nonclinical: heavy drinking	SCRAMx	100%
Roache et al, 2015 [37]	Laboratory	Assess how we can measure alcohol consumption with this technology	61 (61)	Nonclinical: good health	SCRAM-II (study 1) and SCRAMx (studies 2 and 3)	80%
Roache et al, 2019 [38]	Ambulatory	Assess how we can measure alcohol consumption with this technology	30 (30)	Nonclinical: good health	SCRAM-CAM	80%
Rosenberg et al, 2021 [47]	Ambulatory	Assess acceptability, adherence, and feasibility with this technology	5 (5)	Nonclinical: good health	BACtrack	80%
Sakai et al, 2006 [39]	Ambulatory and laboratory	Assess how we can measure alcohol consumption with this technology	44 (44)	Alcohol-dependent and non–alcohol-dependent	SCRAM	100%
Simons et al, 2015 [44]	Ambulatory	Assess how we can measure alcohol consumption with this technology	60 (60)	Nonclinical: good health	WrisTAS-7	80%
Swift et al, 1992 [45]	Laboratory	Assess how we can measure alcohol consumption with this technology	15 (15)	Nonclinical: good health and alcohol-dependent	WrisTAS	80%
Wang et al, 2019 [10]	Ambulatory and laboratory	Assess how we can measure alcohol consumption with this technology	Still recruiting	Nonclinical: good health	Quantac Tally and BACtrack	20%
Wang et al, 2021 [40]	Ambulatory and laboratory	Assess how we can measure alcohol consumption with this technology	25 (25)	Nonclinical: good health	BACtrack and SCRAM-CAM	80%
Webster and Gabler, 2008 [46]	Laboratory	Assess how we can measure alcohol consumption with this technology	15 (15)	Nonclinical: good health	WrisTAS	80%

^a^The numbers in parentheses in this column are the number of participants that were included in each study after drop outs/withdrawals.

^b^MMAT: Mixed Methods Appraisal Tool.

^c^TAM: transdermal alcohol monitoring.

^d^RCT: randomized controlled trial.

^e^CM: contingency management.

^f^BrAC: breath alcohol concentration.

^g^TAC: transdermal alcohol concentration.

^h^PEM: proton-exchange membrane.

^i^IoT: Internet of Things.

### Quality Assessment

All studies apart from 2 (30/32, 94%) met a minimum of 3 out of 5 criteria [10,23] (Table 1). This is due to Alessi et al [23] not providing details about randomization and participant information, and the study by Wang et al [10] was difficult to score owing to incomplete data collection as their study was still ongoing at the time of publication. With the MMAT, exclusion of low–methodological-quality studies is discouraged [53]. The MMAT scores for each study are provided in Multimedia Appendix 5 [10,11,13,23-51]. All the records selected for data extraction were considered to be at low risk of bias. Owing to the nature of many of the studies included, blinding of participants and personnel was not possible; in some studies (3/32, 9%), there were clear differences in demographics or amount of alcohol provided between groups [30,41,44] or incentives provided within contingency management studies [25] where personnel were required to know participant allocation. Similarly, in many studies (17/32, 53%), there was only 1 group of participants all completing the same task, so randomization or blinding was not required or possible [10,13,24,28,31-34,40,42,43,45,46,48-51], or there was only 1 participant [11]. Some studies (16/32, 50%) did not provide clear information on participant data, randomization, incomplete outcome data, and selective reporting, resulting in potential bias because of limited information in the paper [10,11,24,25,29,30,34,40,41,43-46,48-50].

### Accuracy Measures

#### Overview

Of the 32 studies, 19 (59%) explored the accuracy of SCRAM in laboratory or ambulatory settings [13,23-40], 7 (22%) explored the accuracy of WrisTAS in laboratory or ambulatory settings [34,41-46], and 5 (16%) explored the accuracy of a BACtrack device or prototype in laboratory or ambulatory settings [10,29,30,40,47]. Finally, there were six (6/32, 19%) other TAS devices within the included papers: a PEM fuel cell–based wearable alcohol sensor [50], Milo [11], MOX [49], Quantac Tally [10], a wearable IoT sensor [48], and 1 study (1/32, 3%) that did not name the device but simply described it as a TAS throughout [51].

#### Correlations

##### Overview

Approximately 6% (2/32) of the studies reported discrepancies between SCRAM TAC and self-report [23,39], whereas another study (4/32, 3%) reported moderate to high correlations (*r*=0.79 and *P*<.001 [25]; *r*=0.79-0.94 and *P*<.01 [27]; *r*=0.68 and *P*<.001 [36]). Karns-Wright et al [32] found that, when the concordance with any self-reported drinking was examined as a whole, the concordance rate was significantly higher for moderate and heavy TAC (mean 75.91%, SD 15.06%) than for only heavy TAC (mean 73.69%, SD 15.23%; t_29_=2.05; *P*=.04). Most studies that reported on the correlation between TAC and BAC or BrAC data found moderate to strong correlations (weighted correlations between TAC and estimated BAC (eBAC) [26]: *r*=0.54 and *P*<.001; Pearson correlation coefficient range for peak TAC and BrAC [28]: 0.700-0.997; peak TAC and peak estimated BrAC: *r*=0.56 and *P*=.001; TAC and BrAC [29]: *r*=0.60 and *P*<.001; correlation between peak TAC and peak BrAC [31]: *F*_1,73_=160.03 and *P*<.001). However, Sakai et al [39] found varying disagreements between peak TAC and peak BrAC, and Bland-Altman analyses for laboratory and community participants showed varying disagreement between peak and AUC BrAC and TAC data.

##### WrisTAS

Bond et al [41] found that the correlation between the AUC and the unadjusted number of drinks was 0.62, whereas the correlation between the AUC and the adjusted number of drinks was 0.73 (*P*=.04 for the difference between correlation coefficient estimates). A low correlation was reported between WrisTAS TAC and BAC (0.20-0.24; correlation between TAC and eBAC in adolescents only: 0.083-0.10; correlation between TAC and eBAC in young adults only: 0.37-0.39; significantly different by age group: *P*<.001) [42]. However, Swift et al [45] found that the TAS signal was similar in amplitude and time course to the BAC curve (curve peak amplitude: *r*=0.61 and *P*<.02; AUCs of BAC and TAS: *r*=0.91 and *P*<.001) [45], whereas another study (1/32, 3%) found that TAC overestimated BAC levels in the self-dose situation by 86%. By contrast, in the laboratory situation, WrisTAS logged TAC peaks just 0.019 g/dL lower than the mean peak BAC [34].

##### BACtrack

An overall high correlation between self-report and TAC was found (correlation for drinking start time: *r*=0.90 and *P*<.001; AUC for drinking event: *r*=0.7 and *P*=.008) [47]. BACtrack was able to distinguish low and high alcohol doses. There was a high correlation between TAC and BrAC data (*r*=0.77; *P*<.001) [29]. For the study by Wang et al [40], data collection was ongoing at the time of publication, but initial data suggested consistency between TAC and BrAC. Fairbairn et al [30] used SCRAM and BACtrack and found that models for estimating real-time BrAC measurements built with data from the Skyn sensor outperformed similar models built with data from SCRAM. Differences between estimated BrAC and BrAC were 60% higher for models based on data from SCRAM versus BACtrack.

#### Sensitivity and Specificity

##### SCRAM

Studies found that the ability of SCRAM to predict or distinguish levels of alcohol consumption had a significant positive relationship with the amount consumed [23,31,33,37,39]. Dougherty et al [28] plotted receiver operating characteristic curves using the peak TAC to predict the number of drinks consumed. A peak TAC value cutoff of ≥0.011 g/dL classified participants as having drank at least one beer with 97.9% accuracy (AUC=0.99, sensitivity 98.6%, and specificity 95%). A peak TAC value cutoff of ≥0.024 g/dL classified participants as having drank 1-2 beers or >2 beers with 85.1% accuracy (AUC=0.93, sensitivity 92.3%, and specificity 76.2%).

##### WrisTAS

Bond et al [41] found that the sensitivity for WrisTAS compared with self-report was 85.6% and the specificity compared with self-report was 67.5% (percentage of days during which diaries and devices indicated no drinking event). Croff et al [42] found that the sensitivity was 40% and the specificity was 87.9%. Marques and McKnight [34] found that the sensitivity was 24%. This low rate was explained because of erratic output and unreliable data recording during 67% of episodes recorded. WrisTAS more accurately estimated laboratory dosing levels than self-dosing. Simons et al [44] found that WrisTAS correctly identified 85.74% of self-reported drinking (sensitivity 72.35% and specificity 92.94%).

#### Failure Rates

The failure rate for SCRAM was reported to be low at 2% [29] and, in the study by Fairbairn et al [30], 6 files were found to be missing because of procedural issues associated with SCRAM assignment, and 1 was missing because of device malfunction.

Croff et al [42] found that, of the 471,625 data points collected via the WrisTAS, 35,803 data points were missing or corrupted, which equated to 186 whole or partial days of missing data. This was more common for adolescent (11.78% of daily data collected) than for young adult participants (8.59%; *χ*^2^=−18.4; *P*<.001). WrisTAS data files were often found to have spikes despite the participants not reporting a drinking event. For some of these, it was most likely an environmental alcohol (perfume or hand sanitizer). However, other spikes may have been due to unreported alcohol consumption based on the data interpretation [43].

Marques and McKnight [34] found a low sensitivity, the reason being erratic output and not recording or missing data during drinking episodes (this was reported as a chipset rather than a sensor problem). It was also reported that it was harder to interpret the data because of *noisy* patterns. However, Swift et al [45] reported occasional noise, usually because of defects in the interface cable, but transient noise was easily distinguished from a drinking event.

The BACtrack prototype was found to have a higher failure rate between 16% and 38% [29,30]. Missing data were due to device malfunction and device and user issues (described as being lost by the researchers as they learned how to use the prototypes). It was found that BACtrack Skyn data showed a lot of *noise* thought to be due to the rapid increase or decrease of the TAC signal [40].

#### Time Lag

The peak TAC measured by SCRAM was 120 minutes after the peak BrAC [29]. Across all measured portions of the BrAC curve, SCRAM lagged by 69 minutes (*P*<.001). Approximately 9% (3/32) of the studies found a delay in SCRAM data behind alcohol consumption and BrAC data of approximately 2-3 hours [13,35,39] or even longer and mean TAC peak delays of 4.5 (SD 2.9) hours relative to BAC peaks [34].

The mean time to peak for WrisTAS was 71 (SE 7) minutes, and the mean time for the BAC curve was 107 (SE 12) minutes; for the TAS curve, the mean difference in onset times was significantly different (*P*<.02). In the controlled consumption experiment, the peak values of the TAS concentration-time curves lagged by approximately 30 minutes behind the breathalyzer time curves. In intoxicated participants, the peak TAS signal lagged up to 120 minutes behind the BAC.

BACtrack measurements lagged the peak BrAC by 24 minutes (*P*<.001) [29]. Rosenberg et al [47] found a time lag of 2 and 3 hours for peak TAC compared with peak BrAC in low- and high-dose groups, respectively. Initial results found a time lag for Skyn of approximately 135 minutes after drinking for peak TAC compared with 60 minutes for peak BrAC [10].

#### Other TAS Devices

A PEM fuel cell–based wearable alcohol-sensing device was used in a human volunteer pilot study [50]. The measurements from the device showed a significant correlation with the calculated theoretical values. The device provided continuous BAC data, which were processed and fitted into a principal component regression model to determine the accurate transcutaneous alcohol content. Breathalyzer measurements showed greater variation than sensor data.

Lansdorp et al [11] used the Milo sensor to measure data continuously over 2 days. After a state of baseline data was recorded, a solution of 0.05 mol/L ethanol in 1x phosphate-buffered saline was flowed over the diffusion-limiting membrane. The mean sensor response time (time to reach the current 50% of the maximal plateau after the addition of a known concentration of ethanol) under laboratory conditions was 36 (SD 6) minutes with 12 sensors. A linear sensor range between 0 and 0.05 mol/L of ethanol was found.

Investigation of a MOX sensor found that the TAC curve was right-shifted from the BAC and BrAC curves and there was a time delay for the peak of approximately 80 minutes. The 2 different concentrations of ethanol (0.5 g/L and 0.8 g/L) absorbed by the participants could be discriminated [49].

The Quantac Tally was explored before Quantac Co. ceased its business operations. TAC measurements peaked on average 115 minutes after drinking onset, with a gradual increase to peak concentration [40].

The use of a wearable IoT sensor [48] found that the mean values could be distinguished between different alcohol doses. They also compared wearing the sensor on different body parts (left upper arm and left ankle); these results found that the different body parts found different TAC gas (TACg) data. To further explore how perspiration affects the data, the same participant wore the device as normal versus wearing 3 jumpers or jackets to induce sweating. The comparison between BAC and TACg for AUC ratio was 0.98 for BAC and 0.52 for TACg. Spikes in TACg data from the jumper experiment were correlated with spikes in humidity [48].

Davidson et al [51] explored whether a TAS could accurately measure low BAC. The results showed that the device was able to detect TAC at the lowest dose (10 mg/dL), which was not measured by BrAC or BAC. For doses 2 (20 mg/dL) and 3 (40 mg/dL), TAC, BrAC, and BAC were in general agreement. However, BrAC and BAC were in stronger agreement (dose 2: *r*=0.092 and *P*<.001; dose 3: *r*=0.88 and *P*<.001) than TAC compared with BrAC (dose 2: *r*=0.52 and *P*>.05; dose 3: *r*=0.7 and *P*>.05) and BAC (dose 2: *r*=0.60 and *P*<.05; dose 3: *r*=0.52 and *P*>.05). There was also a time lag for TAC output, which was affected by *noise*.

## Discussion

### Principal Findings

The aim of this review was to assess the current knowledge on the accuracy of TAS devices. We identified 32 papers, few of which reported the use of this technology in clinical populations. Of the 32 papers, we identified only 3 (9%) studies that used alcohol-dependent participants. Most studies used either SCRAM (19/32, 59%) or WrisTAS (7/32, 22%). However, there were some studies that investigated BACtrack Skyn (5/32, 16%) and others (6/32, 19%). This is a growing field, and the production and investigation of additional TAS devices supports this. This review found that wearable TAS devices could detect alcohol consumption with moderate to strong accuracy over various periods. However, factors such as the amount of alcohol consumed, the environment (laboratory and self-dose real-world setting), age, and where the device is worn must be considered. The findings differed across the TAS brands included, and studies on each brand reported different limitations; for example, time lag compared with BrAC or BAC, data file errors, or device failure.

The SCRAM, WrisTAS, and BACtrack studies showed reasonably high data capture rates but demonstrate that this is <100% [26,27,32,34,41,42,44]. There were also reports of device failure, malfunctions, noise, and tampering, all of which reduced the amount of successful data capture [29,30,43]. It was noted over time that SCRAM lost accuracy, most likely because of water accumulation [34]. The main feature of using these devices compared with other tests, such as breathalyzers or urine tests, is the capacity for continuous measurements, so this is an important benefit of TAS devices. There has been some investigation into factors such as sex and BMI on episode detection, with no conclusive relationship determined [26,28].

SCRAM devices had the lowest amount of recorded failure rates of the included devices [29,30,34,42], but the time lag to peak alcohol concentration was considerably longer than that of the other brands of TAS devices [10,13,29,34,40,45,47]. Although SCRAM, WrisTAS, and BACtrack showed positive correlations with self-report, BAC, and BrAC, the accuracy of determining the amount of alcohol appeared to be stronger in BACtrack than in SCRAM in some studies [29] but not in others [40]. WrisTAS showed the weakest support for accuracy [34,42]. There were fewer studies on BACtrack compared with SCRAM and WrisTAS, and the studies included that used BACtrack mostly used a device prototype. However, the preliminary results appear promising. Similar to WrisTAS, there seems to be a high failure rate and noise in the data files, but there is support for a shorter time lag compared with SCRAM as well as a strong positive correlation with BAC or BrAC, and it maintains the ability to distinguish between high and low doses [29,40,47].

Approximately 3% (1/32) of the studies compared SCRAM and WrisTAS in laboratory and ambulatory settings. When the participants self-dosed, the true positive rates for both devices increased and, the higher the peak BAC level, the higher the rate of true positive TAC [34]. Although SCRAM more accurately estimated self-dosing and WrisTAS more accurately estimated laboratory dosing, neither was accurately estimated in both settings. This difference between devices is something that should be investigated further and could affect preference for brand of device for different uses in different settings [34].

Our findings relating to SCRAM, WrisTAS, and BACtrack were largely in line with the review findings of van Egmond [15]. Data from these TAS devices were found to moderately to highly positively correlate with self-report and BrAC, with WrisTAS and BACtrack devices showing higher malfunction, failure rates, and noise within data files compared with SCRAM. Van Egmond [15] did not include papers exploring other TAS devices.

There was 1 individual paper (1/32, 3%) on each of the other 6 wearable TAS devices [10,11,48-51]. The development of other devices suggests that this is a rapidly evolving technology. The unnamed device in the study by Davidson et al [51] demonstrates the ability of this technology to accurately measure low BAC and the general agreement between TAC, BAC, and BrAC data. Li et al [48] is the only study that compares data taken from the same device but worn on different body parts. The data found suggest that the location of the device on the user does affect the measurements captured, which is important to consider.

### Limitations

This review highlights the small but growing number of studies investigating the accuracy of wearable TAS devices. With only a small number of studies testing more than one brand or even the same setting, intervention, or population, it is difficult to draw direct comparisons. Most studies (29/32, 91%) included healthy adults, and the length of time studied ranged from a day to a few months. If the ultimate objective is for these devices to be used within the clinical population or the criminal justice system for extended periods, this manner of use needs to be explored further. No included study was conducted in a criminal justice setting with offenders. However, there is a growing appeal for this technology to be used in this environment; for example, the use of SCRAM in the UK criminal justice system for alcohol-related offenses [54].

A mix of study designs was included in both the ambulatory and laboratory settings. Although it is useful to conduct studies in controlled laboratory environments to investigate validity, reliability, and accuracy, the target use is not necessarily a laboratory environment. Hence, how the devices perform in vivo with populations other than those studied must be investigated. This would then be able to inform the devices’ long-term effectiveness in future treatment, interventions, research, and policy.

### Conclusions

There are currently a small number of studies in this area, but research on the use of this technology is growing and, owing to technological advancements, the accuracy and ability of these devices is improving considerably. What is needed is for research to expand into other populations, such as clinical populations and offenders within the criminal justice system, to examine their accuracy and reliability in the intended target populations and contexts. Although the accuracy outcomes for this technology are promising, there is a limit to this research because of the mostly laboratory and short-duration study design.

The use of wearable TAS devices is becoming more accepted and appealing to society, as evidenced by their increasing implementation in the criminal justice system and increasing research. The implications of this review are that we need to investigate the engagement in real-world settings, where transdermal sensors are intended to be implemented, with the target populations. This can then inform clinical interventions, treatment, research, and use.

## Appendix

Multimedia Appendix 1 Database search, screening, and inclusion of studies for transdermal alcohol sensor accuracy systematic review.

Multimedia Appendix 2 Database search terms.

Multimedia Appendix 3 Data extraction form.

Multimedia Appendix 4 Transdermal alcohol concentration and blood alcohol concentration measurement definition.

Multimedia Appendix 5 Risk of bias Mixed Methods Appraisal Tool scores for transdermal alcohol sensor accuracy systematic review.

## Abbreviations

AUC: area under the curve
BAC: blood alcohol concentration
BrAC: breath alcohol concentration
eBAC: estimated blood alcohol concentration
IoT: Internet of Things
MMAT: Mixed Methods Appraisal Tool
NHS: National Health Service
NIHR: National Institute for Health Research
PEM: proton-exchange membrane
PRISMA-P: Preferred Reporting Items for Systematic Reviews and Meta-Analyses Protocols
TAC: transdermal alcohol concentration
TACg: transdermal alcohol concentration gas
TAS: transdermal alcohol sensor
