# Supplementary material for: Accuracy of Wearable Transdermal Alcohol Sensors: Systematic Review
Source: J Med Internet Res. 2022 Apr 14;24(4):e35178. doi: 10.2196/35178 (PMC9052024; doi:10.2196/35178)
Supplement: Multimedia Appendix 1 [file jmir_v24i4e35178_app1.docx]

Multimedia appendix 1. Database search, screening, and inclusion of studies for TAS accuracy systematic review.

| **Title (Author, Year)** | **Included/Excluded** | **Reason** | **Database/Hand search** |
| --- | --- | --- | --- |
| Experiences with SCRAMx alcohol monitoring technology in 100 alcohol treatment outpatients (Alessi et al. 2017) | Excluded | Wrong outcomes | PubMed, Scopus |
| Objective continuous monitoring of alcohol consumption for three months among alcohol use disorder treatment outpatients (Alessi et al. 2019) | Included | - | CINAHL, Scholar, PubMed, Scopus |
| M-estimation in a diffusion model with application to biosensor transdermal blood alcohol monitoring (Allayioti et al. 2020) | Excluded: Full text screen | Wrong intervention | Scholar |
| Sn02 sensors for a portable transdermal alcohol detector via finger (Annaouch et al. 2019) | Excluded: Full text screen | Wrong technology | Scopus |
| Chemi-luminescent visualization system for evaluation of alcohol metabolism based on transdermal emission of gaseous ethanol (Arakawa et al.2012) | Excluded: Title/abstract screen | Wrong intervention | Scopus |
| Contingency Management and Brief Motivational Interviewing Interventions for Impaired Driving Offenders (Averill et al. 2017) | Excluded: Title/abstract screen | Dissertation | Scholar |
| Contingency management for DUI offenders using transdermal alcohol sensors - A pilot randomised controlled study (Averill et al. 2017) | Excluded: Full text screen | Duplicate | Scholar |
| Transdermal alcohol monitoring combined with contingency management for driving while impaired offenders: A pilot randomized controlled study (Averill et al. 2018) | Excluded | Wrong outcomes | CINAHL. Scholar, Ovid, PubMed, Scopus |
| Quantitative determination of caffeine and alcohol in energy drinks and the potential to produce positive transdermal alcohol concentrations in human subjects (Ayala et al. 2009) | Included | - | Ovid, PubMed, Scopus |
| Detecting drinking episodes in young adults using smartphone-based sensors (Bae et al. 2017) | Excluded: Full text screen | Wrong intervention | Scholar |
| The prohorov metric framework and aggregate data inverse problems for random PDEs (Banks et al. 2018) | Excluded: Full text screen | Wrong intervention | Scholar |
| Contingency management for alcohol use reduction - A pilot study using a transdermal alcohol sensor (Barnett et al. 2011) | Included | - | CINAHL, Ovid, PubMed, Scopus |
| Predictors of detection of alcohol use episodes using a transdermal alcohol sensor (Barnett et al. 2014) | Included | - | CINAHL, Ovid, PubMed, Scopus |
| The transdermal alcohol sensor macro (TASMAC)- A rapid data processing tool for use with the SCRAMx alcohol sensor: 726 (Barnett et al. 2015) | Excluded: Title/abstract screen | Abstract | Ovid |
| A preliminary randomized controlled trial of contingency management for alcohol use reduction using a transdermal alcohol sensor (Barnett et al. 2017) | Included | - | CINAHL, Ovid, PubMed, Scopus |
| Clinical and legal aspects of an abstinence control by taking into special consideration of continuous transdermal alcohol monitoring (Beck 2013) | Excluded: Full text screen | Review | Scopus |
| Combining ecological momentary assessment with objective, ambulatory measures of behaviour and physiology in substance-use research (Bertz et al. 2018) | Excluded: Full text screen | Review | Scholar |
| Adjustments for drink size and ethanol content: new results from a self-report diary and transdermal sensor validation study (Bond et al.2014) | Included | - | Scholar, Ovid, PubMed, Scopus |
| The association between negative and positive affect and alcohol use - An ambulatory study (Bresin & Fairbairn 2019) | Excluded: Full text screen | Wrong intervention | CINAHL, Scholar, Scopus |
| Understanding new technologies in today’s car (Bukowski 2016) | Excluded: Full text screen | Grey literature | Scholar |
| Reducing drinking among people experiencing homelessness: protocol for the development and testing of a just-in-time adaptive intervention (Businelle et al. 2020) | Excluded: Full text screen | Protocol | Scholar, PubMed, Scopus |
| Transdermal monitors to assess alcohol consumption in real-time and real-life: a qualitative study on user-experience (Caluzzi et al. 2019) | Excluded | Wrong outcomes | Hand search |
| Surveillance Medicine in the Digital Era: Lessons From Addiction Treatment (Carter et al. 2018) | Excluded: Full text screen | Review | CINAHL |
| Changes in accommodation dynamics after alcohol consumption, for two different doses (Casares-Lopez et al. 2020) | Excluded: Title/abstract screen | Wrong intervention | Scholar |
| Feasibility and reliability of continuously monitoring alcohol use among female adolescents and young adults (Croff et al. 2020) | Included | - | Scholar, Ovid, PubMed, Scopus |
| Using drinking data and pharmacokinetic modelling to calibrate transport model and blind deconvolution based data analysis software for transdermal alcohol biosensors (Dai et al.2016) | Excluded: Full text screen | Wrong intervention | Ovid, PubMed, Scopus |
| Behavioural effects and pharmacokinetics of low-dose intravenous alcohol in humans (Davidson et al. 1997) | Included | - | Ovid, PubMed, Scopus |
| Inside Outside Spring 2015 (Department of Psychology, Uni of Southern California 2015) | Excluded: Title/abstract screen | Wrong intervention | Scholar |
| Comparing the detection of transdermal and breath alcohol concentrations during periods of alcohol consumption ranging from moderate drinking to binge drinking (Dougherty et al. 2012) | Included | - | CINAHL, Ovid, PubMed, Scopus |
| Use of continuous transdermal alcohol monitoring during a contingency management procedure to reduce excessive alcohol use (Dougherty et al. 2014) | Excluded | Wrong outcomes | Scholar, PubMed, Scopus |
| Transdermal alcohol concentration data collected during a contingency management program to reduce at-risk drinking (Dougherty et al. 2015) | Excluded | Wrong outcomes | CINAHL, Scholar, Ovid, PubMed, Scopus |
| Using contingency management procedures to reduce at risk drinking in heavy drinkers (Dougherty et al. 2015) | Excluded: Full text screen | Overlapping sample | Scholar |
| The potential clinical utility of transdermal alcohol monitoring data to estimate the number of alcoholic drinks consumed (Dougherty et al. 2015) | Excluded: Full text screen | Overlapping sample | CINAHL |
| Corrigendum to "Transdermal alcohol concentration data collected during a contingency management program to reduce at-risk drinking (Dougherty et al. 2015) | Excluded: Full text screen | Wrong intervention | CINAHL |
| Corrigendum to “Transdermal alcohol concentration data collected during a contingency management program to reduce at-risk drinking” (Dougherty et al. 2015) | Excluded: Full text screen | Duplicate | Scholar |
| Corrigendum to "Transdermal alcohol concentration data collected during a contingency management program to reduce at-risk drinking (Dougherty et al. 2015) | Excluded: Full text screen | Duplicate | Ovid, PubMed, Scopus |
| Deconvolving blood-alcohol concentration and alcohol beverage consumption from sensor measurements of transdermal alcohol (Dumett et al. 2007) | Excluded: Full text screen | Wrong intervention | Scholar |
| Deconvolving an estimate of breath measured blood alcohol concentration from biosensor collected transdermal ethanol data (Dumett et al. 2008) | Excluded: Full text screen | Wrong intervention | PubMed, Scopus |
| A new paradigm for long-term recovery (Dupont & Humphreys 2011) | Excluded: Full text screen | Editorial | Scholar |
| A multimodal investigation of contextual effects on alcohol's emotional rewards (Fairbairn et al. 2018) | Excluded | Wrong outcomes | CINAHL, Scholar, Scopus |
| Temporal dynamics of transdermal alcohol concentration measured via new generation wrist worn biosensor (Fairbairn & Kang 2019) | Included | - | CINAHL, Scholar, Ovid, PubMed, Scopus |
| Estimating the quantity and time course of alcohol consumption from transdermal alcohol sensor data: A combined laboratory-ambulatory study (Fairbairn et al. 2019) | Included | - | CINAHL, Scholar, Ovid, PubMed, Scopus |
| Understanding social factors in alcohol reward and risk for problem drinking (Fairbairn et al. 2019) | Excluded: Title/abstract screen | Book chapter | Scholar |
| Using machine learning for real time BAC estimation from a new generation transdermal biosensor in the laboratory (Fairbairn et al. 2020) | Included | - | CINAHL, Scholar, Scopus |
| Transdermal alcohol monitors: Research, applications, and future directions (Fairbairn et al. 2021) | Excluded: Full text screen | Book | Scholar |
| Psoriasis and alcohol: is cutaneous ethanol one of the missing links? (Farkas & Kemeny 2010) | Excluded: Title/abstract screen | Wrong intervention | Scholar |
| Rapid evidence review digital interventions to reduce alcohol-related harm- a rapid horizon scanning review (Field et al. 2019) | Excluded: Full text screen | Review | Scholar |
| Behavioural Treatments for Alcohol Use Disorder and Post-Traumatic Stress Disorder (Flanagan et al. 2018) | Excluded: Title/abstract screen | Wrong intervention | Scholar |
| Poly vinyl alcohol hydrogel and its pharmaceutical and biomedical applications- a review (Gajra et al. 2012) | Excluded: Title/abstract screen | Wrong intervention | Scopus |
| A novel non-invasive electrochemical biosensing device for in situ determination of the alcohol content in blood by monitoring ethanol in sweat (Gamella et al. 2014) | Excluded: Full text screen | Wrong intervention | Ovid, PubMed, Scopus |
| Preliminary research informing policy on remote alcohol monitoring in criminal justice: the Scottish experience (Goodall et al. 2016) | Excluded | Wrong outcomes | Scholar |
| Wearable and wireless mHealth technologies for substance use disorder (Goldfine et al. 2020) | Excluded: Full text screen | Review | Scholar |
| Fluorescence-sensing methods (Gryczynski et al. 2003) | Excluded: Title/abstract screen | Wrong intervention | Scopus |
| Use of novel technology-based techniques to improve alcohol-related outcomes in clinical trials (Gurvich et al. 2013) | Excluded: Full text screen | Review | Scopus |
| Transdermal alcohol measurement- a review of the literature (Hawthorne & Wojcik 2006) | Excluded: Full text screen | Review | Scopus |
| Is the concept of compulsion useful in the explanation or description of addictive behaviour and experience? (Heather 2017) | Excluded: Title/abstract screen | Wrong intervention | Scholar |
| Evaluations for NIR spectroscopic transdermal alcohol measurements (Hengfoss et al. 2012) | Excluded: Full text screen | Wrong intervention | Ovid |
| Do variable rates of alcohol drinking alter he ability to use transdermal alcohol monitors to estimate peak breath alcohol and total number of drinks? (Hill-Kapturczak et al. 2014) | Included | - | Scholar |
| Accounting for sex-related differences in the estimation of breath alcohol concentrations using transdermal alcohol monitoring (Hill-Kapturczak et al. 2015) | Excluded | Wrong outcomes | Scholar, Scopus |
| Fluorometric Sniff-Cam (Gas-Imaging System) Utilizing Alcohol Dehydrogenase for Imaging Concentration Distribution of Acetaldehyde in Breath and Transdermal Vapour after Drinking (Iitani et al. 2018) | Excluded: Title/abstract screen | Wrong intervention | Scopus |
| Development and characterization of fuel cell sensor for potential transdermal ethanol sensing (Jalal et al. 2016) | Excluded: Full text screen | Wrong intervention | Scopus |
| Multimodal technique to eliminate humidity interference for specific detection of ethanol (Jalal et al. 2017) | Excluded: Full text screen | Wrong intervention | PubMed, Scopus |
| A wearable micro-fuel cell sensor for the determination of blood alcohol content (BAC): a multivariate regression model approach (Jalal et al. 2020) | Included | - | Scholar |
| Forgotten Histories: Another Road Not Taken-The Charles Merriam-Walter Lippmann Correspondence (Jansen 2010) | Excluded: Title/abstract screen | Wrong intervention | Scholar |
| Characterization of the pharmacokinetics of phosphatidylethanol 16:0/18:1 and 16:0/18:2 in human whole blood after alcohol consumption in a clinical laboratory study (Javors et al. 2016) | Excluded | Wrong outcomes | CINAHL |
| A population-based questionnaire study on the prevalence and epidemiology of burn patients in Denizli, Turkey (Kara et al. 2008) | Excluded: Title/abstract screen | Wrong intervention | Scholar |
| Time delays in transdermal alcohol concentrations relative to breath alcohol concentrations (Karns-Wright et al. 2017) | Included | - | CINAHL, Scholar, Ovid, PubMed, Scopus |
| The correspondence between transdermal alcohol monitoring and daily self-reported alcohol consumption (Karns-Wright et al. 2018) | Included | - | Scholar |
| The best of both worlds: Avatar-assisted therapy offers the benefits of therapist-assisted and Internet-based interventions (Kendzor & Herbert 2017) | Excluded: Title/abstract screen | Wrong intervention | Scholar |
| Validation of transdermal alcohol concentration data collected using wearable alcohol monitors: A systematic review and meta-analysis (Kianersi et al. 2020) | Excluded: Full text screen | Review | CINAHL, Scholar, Ovid, PubMed, Scopus |
| Efficacy of Frequent Monitoring With Swift, Certain, and Modest Sanctions for Violations: Insights From South Dakota’s 24/7 Sobriety Project (Kilmer et al. 2013) | Excluded | Wrong outcomes | Hand search |
| Essays on the consequences of drug use and drug testing (Kilmer 2007) | Excluded: Title/abstract screen | Dissertation | Scholar |
| Noninvasive alcohol monitoring using a wearable tattoo-based iontophoretic-biosensing system (Kim et al. 2016) | Excluded: Full text screen | Wrong intervention | Scopus |
| Alternative sampling strategies for the assessment of alcohol intake of living persons (Kummer et al. 2016) | Excluded: Title/abstract screen | Dissertation | Scholar |
| Alternative sampling strategies to monitor alcohol consumption in case of driver's licence regranting (Kummer 2016) | Excluded: Full text screen | Review | Scholar |
| Wearable enzymatic alcohol biosensor (Lansdorp et al. 2019) | Included | - | PubMed, Scopus |
| Transdermal alcohol measurements using MOX sensors in clinical trials (Lawson et al. 2017) | Excluded | Conference | Hand search |
| Skin alcohol perspiration measurements using MOX sensors (Lawson et al. 2019) | Included | - | Scopus |
| Continuous objective monitoring of alcohol use: twenty-first century measurement using transdermal sensors (Leffingwell et al. 2013) | Excluded: Full text screen | Review | Scopus |
| Comparing a distributed parameter model-based system identification technique with more conventional methods for inverse problems (Li et al. 2019) | Excluded: Full text screen | Wrong intervention | Scholar, PubMed |
| A discreet wearable IoT sensor for continuous transdermal alcohol monitoring - challenges and opportunities (Li et al. 2020) | Included | - | Scopus |
| Determining blood and:or breath alcohol concentration from transdermal alcohol data (Luczak et al. 2013) | Excluded: Full text screen | Conference | Scopus |
| Development of a real-time repeated-measures assessment protocol to capture change over the course of a drinking episode (Luczak et al. 2015) | Included | - | CINAHL, Scholar, Ovid, PubMed, Scopus |
| Obtaining continuous BrAC/BAC estimates in the field: A hybrid system integrating transdermal alcohol biosensor, Intellidrink smartphone app, and BrAC estimator software tools (Luczak et al. 2018) | Excluded: Full text screen | Wrong intervention | CINAHL, Scholar, Ovid, PubMed |
| Estimating BrAC from transdermal alcohol concentration data using the BrAC estimator software program (Luczak & Rosen 2014) | Excluded: Full text screen | Wrong outcomes | CINAHL, Scholar, Ovid, PubMed, Scopus |
| Special issue on alcohol biosensors: Development, use, and state of the field: Summary, conclusions, and future directions (Luczak & Ramchandani 2019) | Excluded: Full text screen | Review | PubMed |
| Technologies to Monitor the Behaviour of Alcohol-Involved Drivers (Marques 2011) | Excluded: Title/abstract screen | Book chapter | Scholar |
| Field and laboratory alcohol detection with 2 types of transdermal devices (Marques & McKnight 2009) | Included | - | Ovid, PubMed, Scopus |
| Translating transdermal alcohol monitoring procedures for contingency management among adults recently arrested for DWI (Mathias et al. 2018) | Excluded | Wrong outcomes | Scholar, Ovid, PubMed |
| Transdermal alcohol monitoring - case studies (McKnight et al. 2012) | Excluded: Full text screen | Report | Scholar |
| Behavioural impulsivity does not predict naturalistic alcohol consumption or treatment outcomes (Mullen et al. 2016) | Excluded: Full text screen | Wrong intervention | Scholar |
| Patients' Experiences of Using a Cellular Photo Digital Breathalyzer for Treatment Purposes (Nehlin et al. 2018) | Excluded: Title/abstract screen | Wrong intervention | Scholar |
| An Experimental Trial Exploring the Impact of Continuous Transdermal Alcohol Monitoring upon Alcohol Consumption in a Cohort of Male Students (Neville et al. 2013) | Excluded | Wrong outcomes | Hand search |
| Transdermal anti-inflammatory activity of bilayer film containing olive compound hydroxytyrosol: physical assessment, in vivo dermal safety and efficacy study in Freund's adjuvant-induced arthritic rat model (Ng et al. 2017) | Excluded: Title/abstract screen | Wrong intervention | Scopus |
| Combining transdermal and breath alcohol assessments, real-time drink logs and retrospective self-reports to measure alcohol consumption and intoxication across a multi-day music festival (Norman et al. 2020) | Included | - | Ovid, PubMed, Scopus |
| Contingency management treatment for substance use disorders: How far has it come, and where does it need to go? (Petry et al. 2017) | Excluded: Title/abstract screen | Wrong intervention | Scholar |
| Evaluation of the alcopatch, a transdermal dosimeter for monitoring alcohol consumption (Phillips et al. 1995) | Excluded: Title/abstract screen | Wrong intervention | Scopus |
| Assessment of alcohol use in the natural environment (Piasecki 2019) | Excluded: Full text screen | Review | CINAHL, Scholar, Ovid, PubMed, Scopus |
| Ongoing monitoring of alcohol use tied to clear consequences- An evidence-based strategy for enhancing the likelihood of sustained sobriety (Pigot year unknown) | Excluded: Full text screen | Review | Scholar |
| Considerations for Implementing Contingency Management in Substance Abuse Treatment Clinics: The Veterans Affairs Initiative as a Model (Rash & DePhilippis 2019) | Excluded: Title/abstract screen | Wrong intervention | Scholar |
| Monitoring alcohol use in heavy drinking soup kitchen attendees (Rash et al. 2019) | Included | - | CINAHL, Scholar, PubMed, Scopus |
| Development and validation of a scale measuring attitudes toward non-drinkers (Regan & Morrison 2011) | Excluded: Title/abstract screen | Wrong intervention | Scholar |
| Mobile alcohol biosensors and pharmacotherapy development research (Roberts & McKee 2019) | Excluded: Full text screen | Review | PubMed, Scopus |
| Continuous Transdermal Alcohol Monitoring: A practitioner’s guide (Robertson et al. 2007) | Excluded: Title/abstract screen | Grey literature | Scholar |
| Using transdermal alcohol monitoring to detect low-level drinking (Roache et al. 2015) | Included | - | Ovid, PubMed |
| Processing transdermal alcohol concentration (TAC) data to detect low-level drinking (Roache et al. 2019) | Included | - | CINAHL, Scholar, Ovid, PubMed, Scopus |
| Blind deconvolution for distributed parameter systems with unbounded input and output and determining blood alcohol concentration from transdermal biosensor Data (Rosen et al. 2014) | Excluded: Full text screen | Wrong intervention | PubMed, Scopus |
| Wearable alcohol monitors for alcohol use data collection among college students: feasibility and acceptability in a pilot study (Rosenberg et al. 2021) | Included | - | Hand search |
| Validity of transdermal alcohol monitoring: fixed and self-regulated dosing (Sakai et al. 2006) | Included | - | CINAHL, Ovid, PubMed, Scopus |
| Effects of stomach content on the breath alcohol concentration-transdermal alcohol concentration relationship (Saldich et al. 2021) | Excluded | Wrong outcomes | Hand search |
| Quantifying alcohol consumption: Self-report, transdermal assessment, and prediction of dependence symptoms (Simons et al. 2015) | Included |  | CINAHL, Scholar, Ovid, PubMed, Scopus |
| Cardiovascular comorbidity in psoriasis (Singh & Aneja 2011) | Excluded: Title/abstract screen | Wrong intervention | Scholar |
| Deconvolving the input to random abstract parabolic systems: a population model-based approach to estimating blood/breath alcohol concentration from transdermal alcohol biosensor data (Sirlanci et al. 2018) | Excluded: Full text screen | Wrong intervention | Scholar, PubMed, Scopus |
| Estimating the distribution of random parameters in a diffusion equation forward model for a transdermal alcohol biosensor (Sirlanci et al. 2019) | Excluded: Full text screen | Wrong intervention | PubMed |
| Applying a novel population-based model approach to estimating breath alcohol concentration (BrAC) from transdermal alcohol concentration (TAC) biosensor data (Sirlanci et al. 2019) | Excluded: Full text screen | Wrong intervention | CINAHL, Scholar, Ovid, PubMed, Scopus |
| Design and Development of Smart SCRAM Device for Effective Ethanol Remote Bio-Monitoring (Suganya et al. 2020) | Excluded: Title/abstract screen | Conference | Scopus |
| Studies on a wearable, electronic, transdermal alcohol sensor (Swift et al. 1992) | Included | - | Ovid, PubMed, Scopus |
| Transdermal alcohol measurement for estimation of blood alcohol concentration (Swift 2000) | Excluded: Full text screen | Wrong intervention | Scopus |
| Transdermal alcohol detection vs. self report for determining alcohol drinking (Swift et al. 2001) | Excluded | Abstract | Scopus |
| Diagnostic characteristics and application of alcohol biomarkers (Topic & Djukic 2013) | Excluded: Full text screen | Review | Scopus |
| Wearable transdermal alcohol monitors: A systematic review of detection validity, and relationship between transdermal and breath alcohol concentration and influencing factors (van Egmond et al. 2020) | Excluded: Full text screen | Review | CINAHL, Scholar, Ovid, PubMed, Scopus |
| A multimodal, longitudinal investigation of alcohol's emotional rewards and drinking over time in young adults (Venerable et al. 2020) | Excluded | Wrong outcomes | Scholar |
| Facilitators and barriers to a contingency management alcohol intervention involving a transdermal alcohol sensor (Villalba et al. 2020) | Excluded | Wrong outcomes | PubMed, Scopus |
| Wrist-worn alcohol biosensors: Strengths, limitations and future directions (Wang et al. 2019) | Included | - | Scholar, PubMed, Scopus |
| Wrist-worn alcohol biosensors: Applications and usability in behavioural research (Wang et al. 2021) | Included | - | Hand search |
| Assessment of dermal ethanol emission sensors: Experimental design (Webster & Gabler 2007) | Excluded: Full text screen | Wrong intervention | Scopus |
| Feasibility of transdermal ethanol sensing for the detection of intoxicated drivers (Webster & Gabler 2007) | Excluded: Full text screen | Conference | Ovid, PubMed, Scopus |
| Modelling of transdermal transport of alcohol - Effect of body mass and gender (Webster & Gabler 2008) | Included | - | Ovid, PubMed |
| Inequities in co-coverage of Preventive Intervention for Children- Analyses of DHS data from low- and Middle Income Countries (Wehrmeister et al. 2015) | Excluded: Title/abstract screen | Wrong intervention | Scholar |
| Enabling personal alcohol tracking using transdermal sensing wristbands (You et al. 2019) | Excluded: Full text screen | Preliminary study design | Scopus |
| 51st Annual proceedings association for the advancement of automative medicine (2014) | Excluded: Title/abstract screen | Conference | Scopus |
| 4th International conference on multi-functional materials and structures, MFMS 2013 (2013) | Excluded: Title/abstract screen | Conference | Scopus |
