# Supplementary material for: Accuracy of Wearable Transdermal Alcohol Sensors: Systematic Review
Source: J Med Internet Res. 2022 Apr 14;24(4):e35178. doi: 10.2196/35178 (PMC9052024; doi:10.2196/35178)
Supplement: Multimedia Appendix 2 [file jmir_v24i4e35178_app2.docx]

Multimedia appendix 2. Database search terms.

|  | **CINAHL was searched via EBSCO. The database coverage was 1937 to present and the database was searched on the 5^th^ February 2021.** | **Search results** |
| --- | --- | --- |
| **1.** | Transdermal alcohol sensor | 12 |
| **2.** | Transdermal alcohol bracelet | 2 |
| **3.** | Transdermal alcohol wristband | 0 |
| **4.** | Transdermal alcohol ankle | 1 |
| **5.** | TI ("transdermal alcohol sensor" or "transdermal alcohol bracelet" or "transdermal alcohol wristband" or "transdermal alcohol ankle") or AB ("transdermal alcohol sensor" or "transdermal alcohol bracelet" or "transdermal alcohol wristband" or "transdermal alcohol ankle") | TI: 5, 0, 0, 0  AB: 10, 0, 0, 1 |
| **6.** | (MH "transdermal alcohol concentration") | 0 |
| **7.** | TI (transdermal alcohol concentration) or AB (transdermal alcohol concentration) | TI: 9 AB: 20 |
| **8.** | 1 or 2 or 3 or 4 or 5 or 6 or 7 | 32 |
| **9.** | (Transdermal alcohol validity) | 2 |
| **10.** | (Transdermal alcohol acceptability) | 0 |
| **11.** | (Transdermal alcohol feasibility) | 2 |
| **12.** | TI (transdermal alcohol validity or transdermal alcohol acceptability or transdermal alcohol feasibility) or AB (transdermal alcohol validity or transdermal alcohol acceptability or "transdermal alcohol feasibility) | TI: 2, 0, 0  AB: 1, 0, 2 |
| **13.** | 9 or 10 or 11 or 12 | 4 |
| **14.** | 7 and 13 | 3 |

|  | **EMBASE, MEDLINE and PscyhINFO were searched via Ovid SP. The database coverage was 1947 to present and the database was searched on the 1^st^ of February 2021.** | **Search results** |
| --- | --- | --- |
| **1.** | exp “Transdermal alcohol sensor”/ | 0 |
| **2.** | Transdermal alcohol sensor.tw | 11 |
| **3.** | Transdermal alcohol bracelet.tw | 1 |
| **4.** | Transdermal alcohol wristband.tw | 0 |
| **5.** | Transdermal alcohol ankle.tw | 0 |
| **6.** | 1 or 2 or 3 or 4 or 5 | 12 |
| **7.** | exp “Transdermal alcohol concentration”/ | 6 |
| **8.** | Transdermal alcohol concnentration.tw | 27 |
| **9.** | 7 or 8 | 27 |
| **10.** | Transdermal alcohol ADJ3 validity.tw | 1 |
| **11.** | Transdermal alcohol ADJ3 acceptability | 0 |
| **12.** | Transdermal alcohol ADJ3 feasibility.tw | 0 |
| **13.** | 10 or 11 or 12 | 1 |
| **14.** | 6 and 9 and 13 | 0 |

|  | **Google Scholar database was searched on the 8^th^ February 2021.** | **Search results** |
| --- | --- | --- |
| **1.** | “Transdermal alcohol sensor device” | 2 |
| **2.** | “Transdermal alcohol bracelet” | 4 |
| **3.** | “Transdermal alcohol wristband” | 0 |
| **4.** | “Transdermal alcohol ankle” | 2 |
| **5.** | “Transdermal alcohol sensor data” | 17 |
| **6.** | “Transdermal alcohol” ADJ3 validity | 4 |
| **7.** | “Transdermal alcohol” ADJ3 acceptability | 2 |
| **8.** | “Transdermal alcohol” ADJ3 feasibility | 2 |
| **9.** | “Transdermal alcohol monitoring devices” | 11 |
| **10.** | “Transdermal alcohol concentration data” | 51 |

|  | **The PubMed database coverage was from 1996 to present and the database was searched on the 2^nd^ February 2021.** | **Search results** |
| --- | --- | --- |
| **1.** | “Transdermal alcohol sensor” [MeSH terms] | 0 |
| **2.** | "Transdermal alcohol" sensor [title/abstract] | 25 |
| **3.** | "Transdermal alcohol" bracelet [title/abstract] | 0 |
| **4.** | "Transdermal alcohol" wristband [title/abstract] | 4 |
| **5.** | "Transdermal alcohol" ankle [title/abstract] | 4 |
| **6.** | 1 or 2 or 3 or 4 or 5 | 26 |
| **7.** | "Transdermal alcohol concentration" [MeSH terms] | 0 |
| **8.** | "Transdermal alcohol concentration" [title/abstract] | 31 |
| **9.** | 7 or 8 | 31 |
| **10.** | 6 and 9 | 12 |
| **11.** | "Transdermal alcohol" validity [tiab] | 9 |
| **12.** | "Transdermal alcohol" acceptability [tiab] | 3 |
| **13.** | "Transdermal alcohol" feasibility [tiab] | 7 |
| **14.** | 11 or 12 or 13 | 17 |
| **15.** | 10 and 14 | 4 |

|  | **Scopus was searched via Elsevier. The database coverage was 1996 to present and the database was searched on the 3^rd^ of February 2021.** | **Search results** |
| --- | --- | --- |
| **1.** | Title/abs/key: Transdermal alcohol sensor | 48 |
| **2.** | Transdermal alcohol wristband | 4 |
| **3.** | Transdermal alcohol bracelet | 7 |
| **4.** | Transdermal alcohol ankle | 9 |
| **5.** | Title: transdermal alcohol concentration | 18 |
| **6.** | 1 or 2 or 3 or 4 | 56 |
| **7.** | Title/ab/keywords: "Transdermal alcohol" validity | 12 |
| **8.** | Title/ab/keywords: "Transdermal alcohol" acceptability | 3 |
| **9.** | Title/ab/keywords: "Transdermal alcohol" feasibility | 7 |
| **10.** | 7 or 8 or 9 | 20 |
| **11.** | 6 and 10 | 14 |
