# Supplementary material for: Accuracy of Wearable Transdermal Alcohol Sensors: Systematic Review
Source: J Med Internet Res. 2022 Apr 14;24(4):e35178. doi: 10.2196/35178 (PMC9052024; doi:10.2196/35178)
Supplement: Multimedia Appendix 3 [file jmir_v24i4e35178_app3.docx]

Multimedia appendix 3. Data extraction form.

| General information | Date of data extraction |
| --- | --- |
|  | Author |
|  | Title |
|  | Year |
|  | Citation |
|  | Country |
|  | Source of funding |
| Study characteristics | Aim |
|  | Design |
|  | Inclusion criteria |
|  | Exclusion criteria |
|  | Recruitment procedure |
| Intervention | Setting |
|  | Intervention |
|  | Control |
|  | TAS device |
| Participant characteristics | Age |
|  | Gender |
|  | Ethnicity |
|  | Health Status |
|  | Clinical or non-clinical |
|  | Number of participants in each group |
| Outcomes and results | Analysis |
|  | Statistical technique |
|  | Primary outcome (definition, measurement tool, length of follow up/times) |
|  | Secondary outcome (definition, measurement tool, length of follow up/times) |
|  | Results (primary) |
|  | Results (secondary) |
|  | Comparison (if any) |
| Feasibility findings | Compliance |
|  | Usability |
|  | Participant burden |
|  | Participant feedback |
|  | Staff/researcher feedback |
|  | Cost to participant |
|  | Incentive/reimbursement |
| Other findings | Implications |
|  | Limitations |
|  | Comparison measure |
|  | Conflicts of interest |
|  | Other |
