# Supplementary material for: Accuracy of Wearable Transdermal Alcohol Sensors: Systematic Review
Source: J Med Internet Res. 2022 Apr 14;24(4):e35178. doi: 10.2196/35178 (PMC9052024; doi:10.2196/35178)
Supplement: Multimedia Appendix 4 [file jmir_v24i4e35178_app4.docx]

**Multimedia appendix 4: TAC and BAC measurement definitions.**

**Transdermal alcohol concentration measurement definition.**

Of those studies who described their measurement of a drinking episode, most used the definition: TAC greater than or equal to 0.02g/dl, with a max absorption rate of 0.05% or a max elimination rate of either 0.003% to <0.025% for peak TAC<=0.015g/dl, or 0.003% to <0.035% for peak TAC>0.015 g/dl [25,27-29,39].

Drinking episodes were defined as passing the threshold of TAC criteria (greater than or equal to 0.03g/dl) across each week of contingency condition and by % days of any drinking measured by any positive TAC reading. Sequence and carry over effects (any carryover effects of CM when the contingency changed) [14,39].

The effect of the CM condition was measured as the % of participants that maintained TAC less than 0.03g/dl per week [14].

Within Karns-Wright et al., possible drinking detection was classified into drinking categories with these definitions: No alcohol (>1 point but <2 TAC points above zero), Heavy TAC (2 or more readings >0.02 g/dl), Moderate (≥3 TAC points above 0 and ≥1 TAC point above 0.01 g/dl but <2 points above 0.02 g/dl), Low (3 or more TAC points > 0, but no points > 0.01 g/dl). The interest of this study was to detect low-level drinking [34].

Simulated alcohol binge was based on the definition of NIAAA [55] to produce a BAC of greater or equal than 0.08 g/dl. This definition of a binge roughly corresponds to at least 5 drinks for men and 4 drinks for women consumed with a 2 hour period [30,55].

**Blood alcohol concentration measurement definition.**

In 2004 the NIAAA defined a binge as a pattern of drinking associated with producing a blood alcohol concentration (BAC) of at least 80 mg/dl (0.08%) [55].
