# Supplementary material for: Accuracy of Wearable Transdermal Alcohol Sensors: Systematic Review
Source: J Med Internet Res. 2022 Apr 14;24(4):e35178. doi: 10.2196/35178 (PMC9052024; doi:10.2196/35178)
Supplement: Multimedia Appendix 5 [file jmir_v24i4e35178_app5.docx]

Multimedia appendix 5. RoB MMAT scores for TAS accuracy systematic review.

| **Study ID** | **Author, Year** | **Total score** | **Criteria 1** | **Criteria 2** | **Criteria 3** | **Criteria 4** | **Criteria 5** |
| --- | --- | --- | --- | --- | --- | --- | --- |
| **Quantitative RCTs design** | | | **Is the randomization appropriate?** | **Are the groups comparable at baseline?** | **Are there complete outcome data?** | **Are outcome assessors blinded to the intervention provided?** | **Did the participants adhere to the assigned intervention?** |
| **1** | Alessi et al. 2019 | 40% | ? | ? | X | X | X |
| **5** | Barnett et al. 2017 | 80% | X | X | X | X | X |
| **8** | Bond et al. 2014 | 60% | ? | X | X | X | X |
| **16** | Fairbairn & Kang 2019 | 60% | X | ? | X | X | X |
| **17** | Fairbairn et al. 2020 | 60% | X | ? | X | X | X |
| **Quantitative non-RCTs design** | | | **Are the participants representative of the target population?** | **Are measurements appropriate regarding both the outcome and intervention?** | **Are there complete outcome data?** | **Are the confounders accounted for in the design and analysis?** | **During the study period, is the intervention administered as intended?** |
| **4** | Ayala et al. 2009 | 80% | ? | X | X | X | X |
| **6** | Barnett et al. 2014 | 100% | X | X | X | X | X |
| **7** | Barnett et al. 2011 | 80% | X | X | X | X | X |
| **10** | Croff et al. 2020 | 80% | X | X | X | X | X |
| **11** | Davidson et al. 1997 | 80% | ? | X | X | X | X |
| **12** | Dougherty et al. 2012 | 100% | X | X | X | X | X |
| **18** | Fairbairn et al. 2019 | 100% | X | X | X | X | X |
| **20** | Hill-Kapturczak et al. 2014 | 80% | ? | X | X | X | X |
| **22** | Jalal et al. 2020 | 60% | ? | X | X | ? | X |
| **24** | Karns-Wright et al. 2018 | 80% | ? | X | X | X | X |
| **25** | Karns-Wright et al. 2017 | 80% | ? | X | X | X | X |
| **26** | Lansdorp et al. 2019 | 60% | X | X | X | ? | X |
| **27** | Lawson et al. 2019 | 60% | ? | X | X | ? | X |
| **28** | Li et al. 2020 | 80% | ? | X | X | X | X |
| **29** | Luczak et al. 2015 | 80% | ? | X | X | X | X |
| **30** | Marques & McKnight 2009 | 60% | ? | X | X | ? | X |
| **33** | Norman et al. 2020 | 60% | ? | X | X | ? | X |
| **34** | Rash et al. 2019 | 100% | X | X | X | X | X |
| **35** | Roache et al. 2015 | 80% | ? | X | X | X | X |
| **36** | Roache et al. 2019 | 80% | ? | X | X | X | X |
| **37** | Rosenberg et al. 2021 | 80% | ? | X | X | X | X |
| **38** | Sakai et al. 2006 | 100% | X | X | X | X | X |
| **40** | Simons et al. 2015 | 80% | ? | X | X | X | X |
| **41** | Swift et al. 1992 | 80% | ? | X | X | X | X |
| **44** | Wang et al. 2019 | 20% | ? | X | X | ? | ? |
| **45** | Wang et al. 2021 | 80% | ? | X | X | X | X |
| **46** | Webster & Gabler 2008 | 80% | ? | X | X | X | X |

X = Yes

X = No

? = Can’t tell
